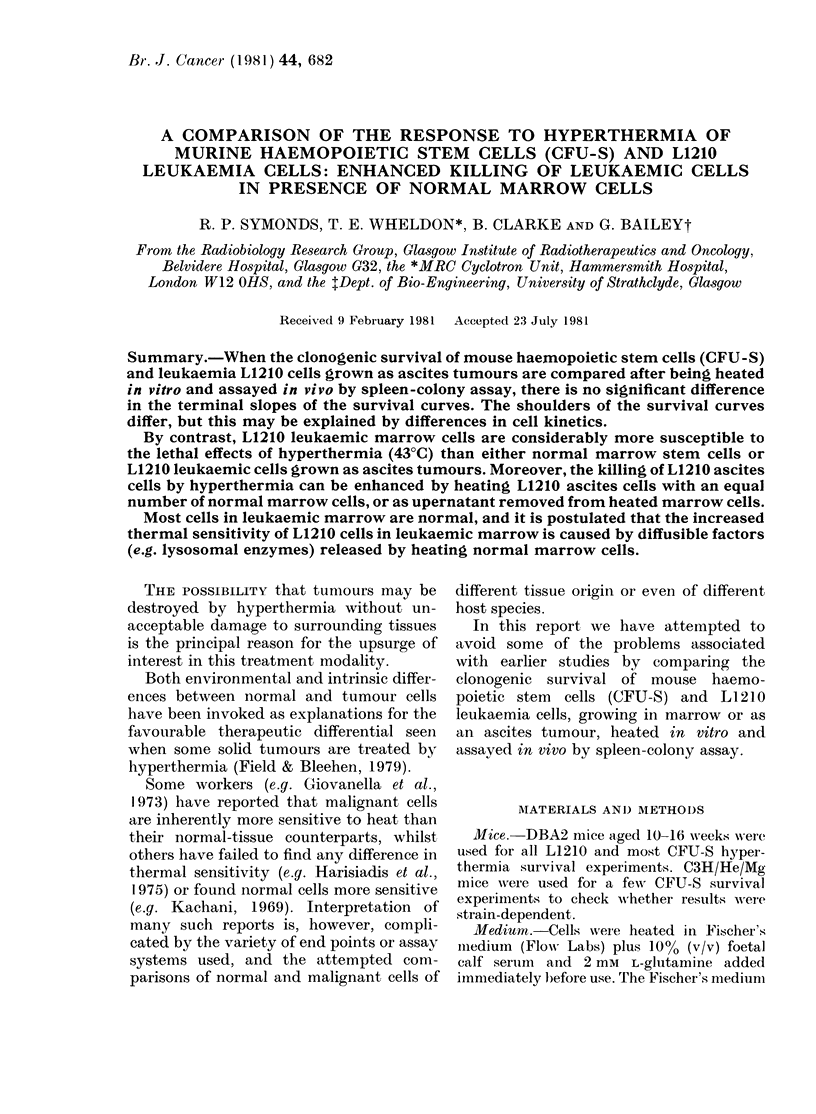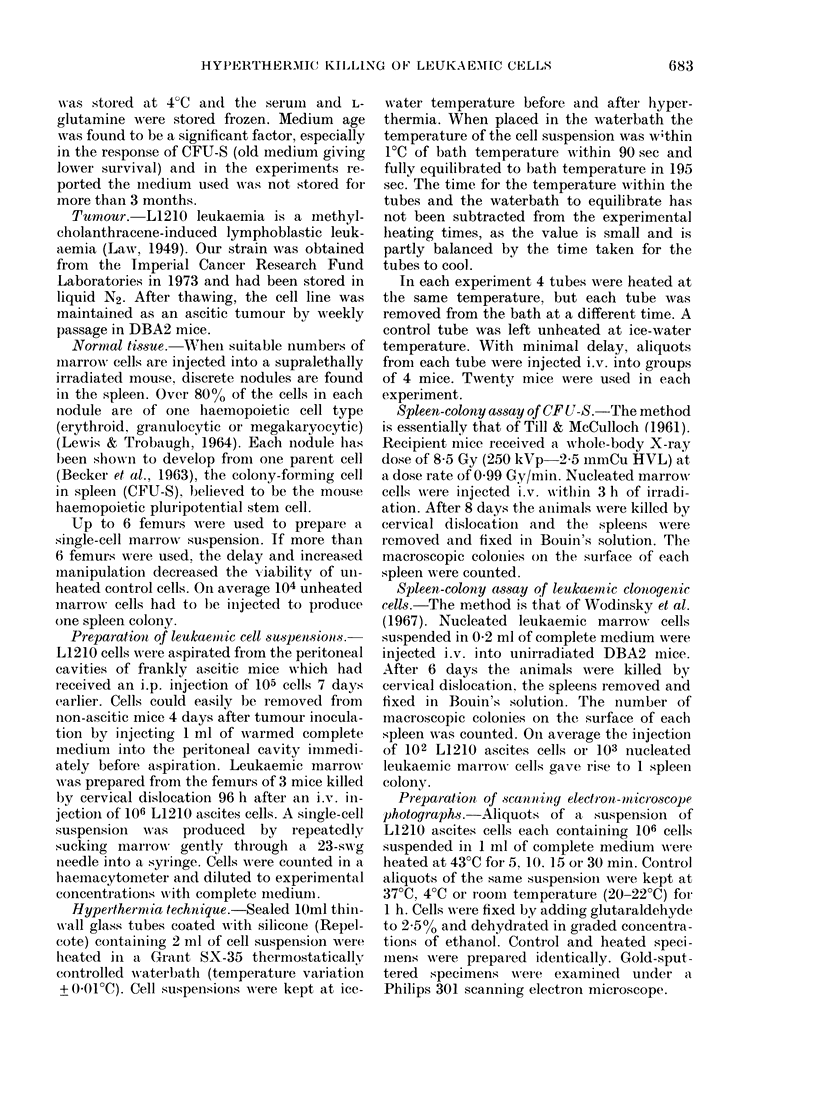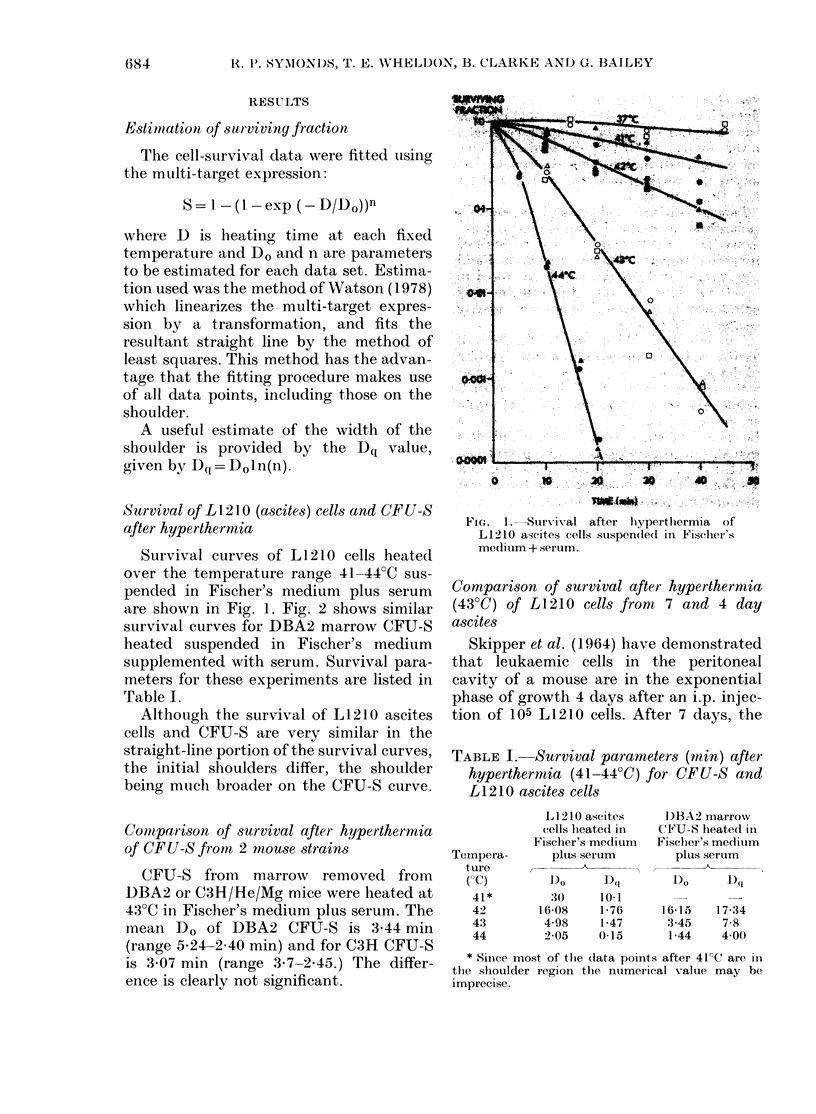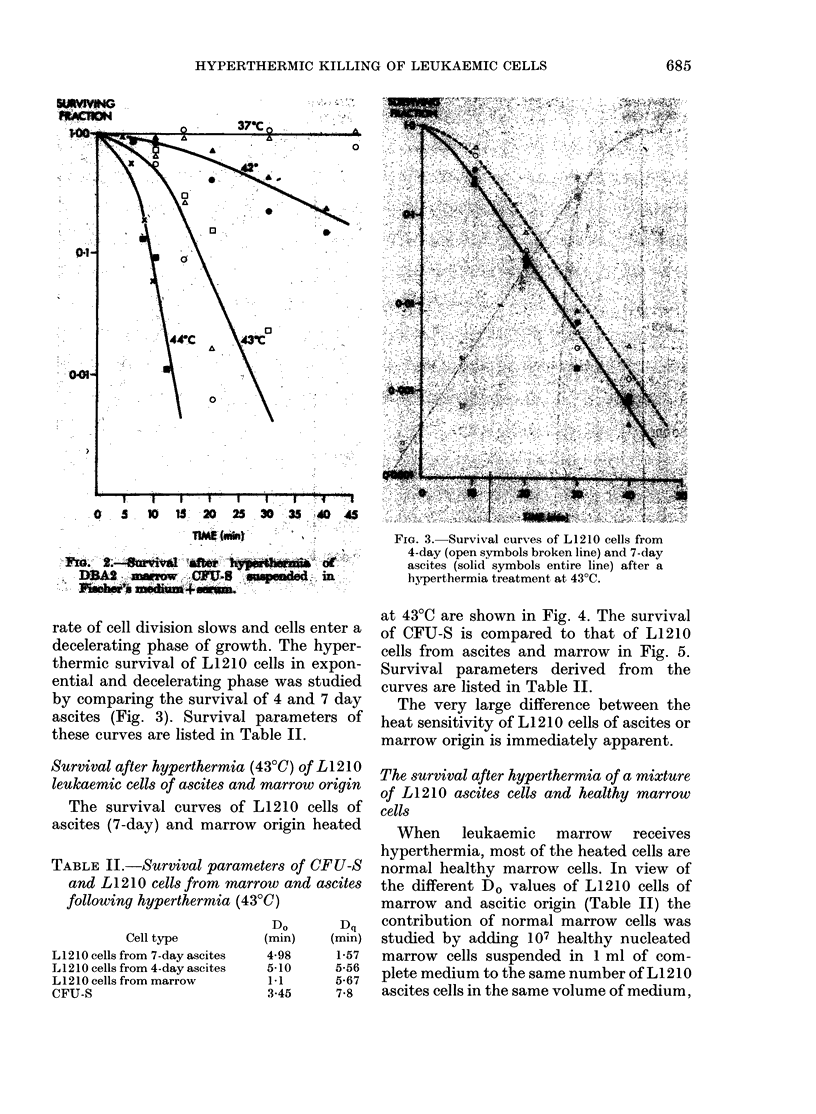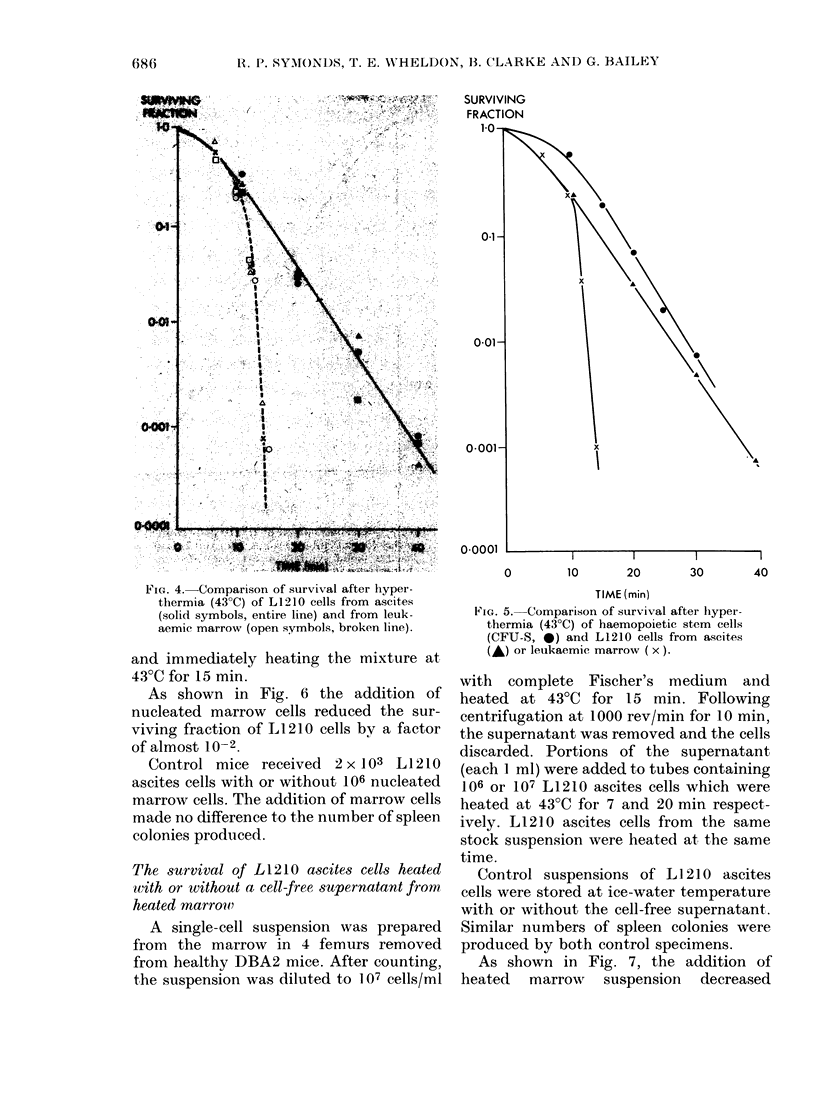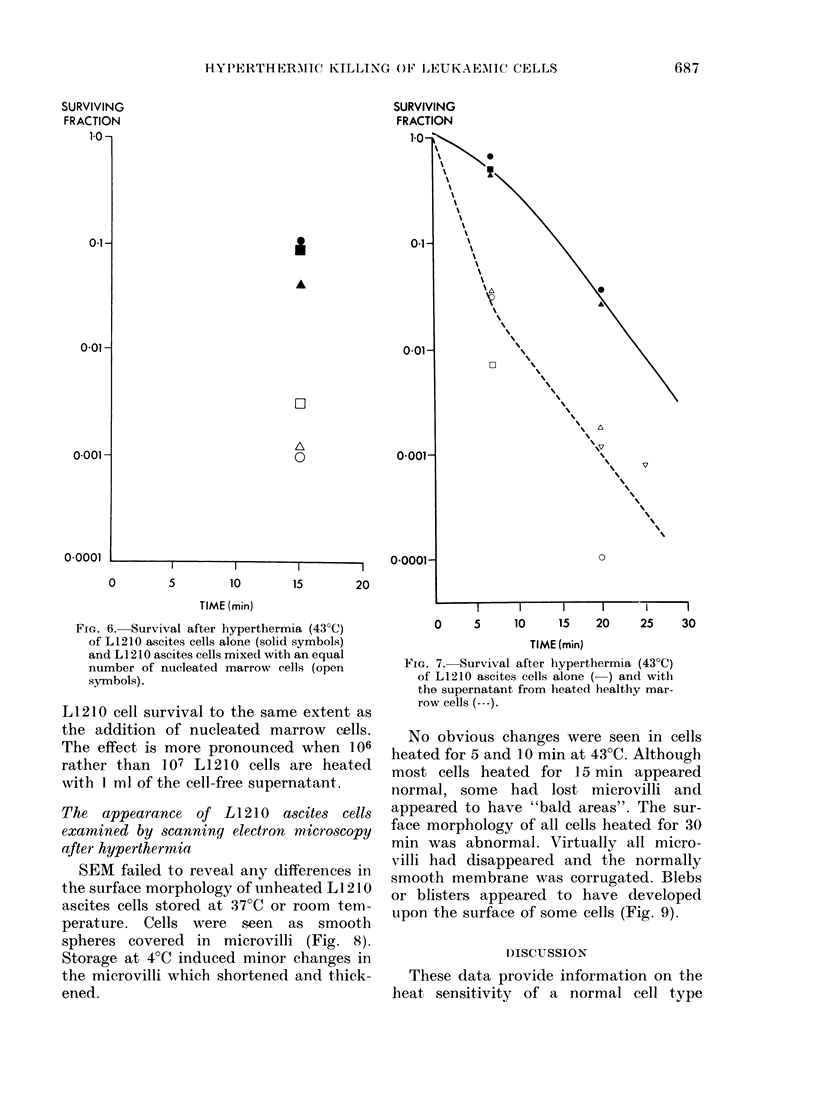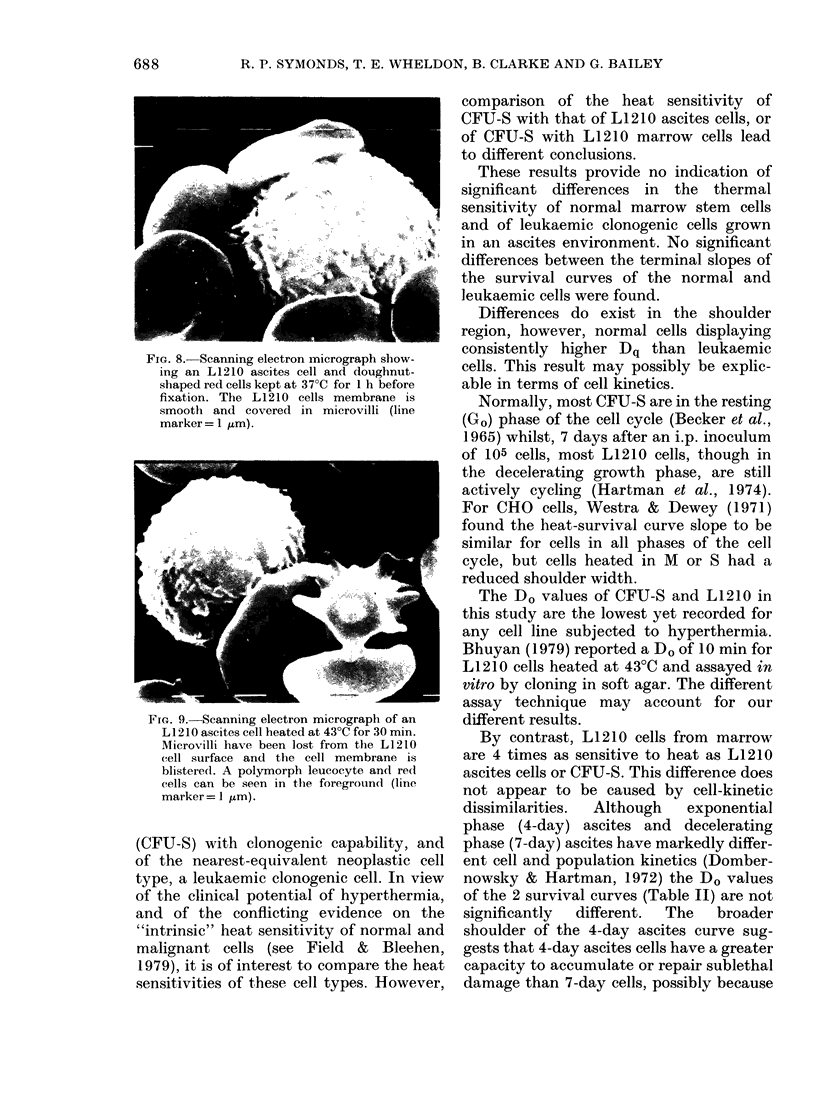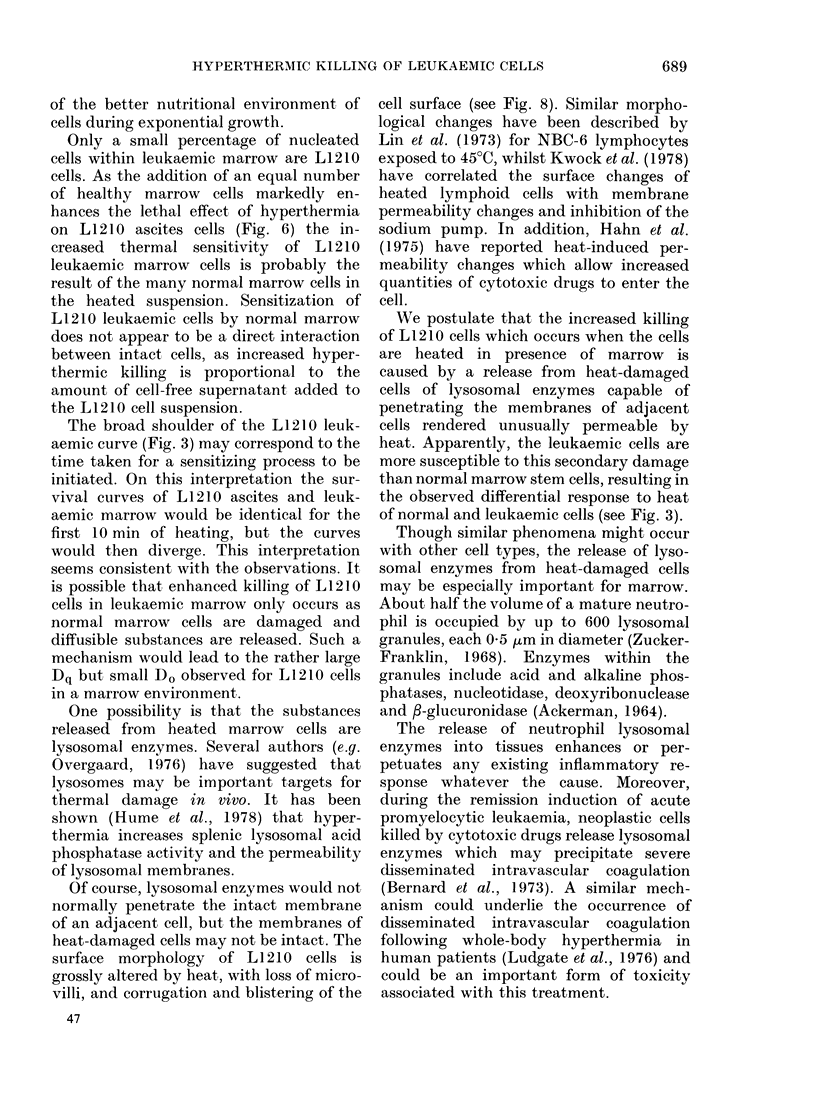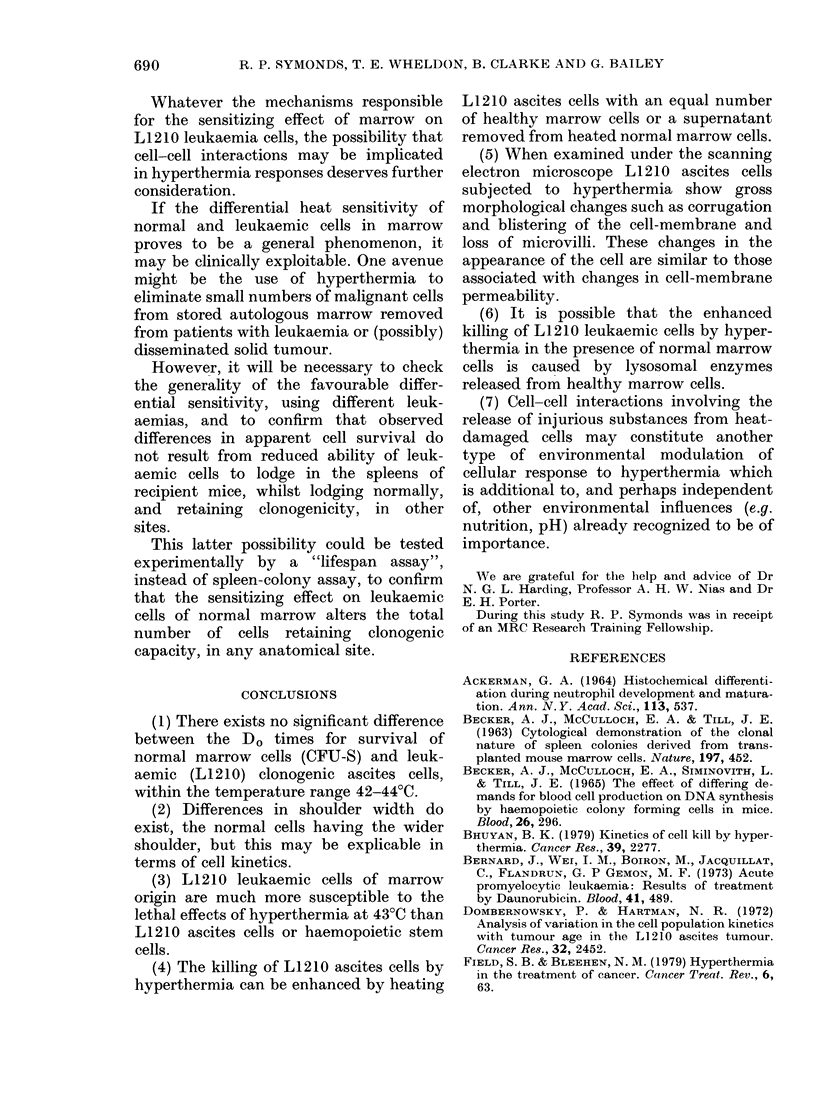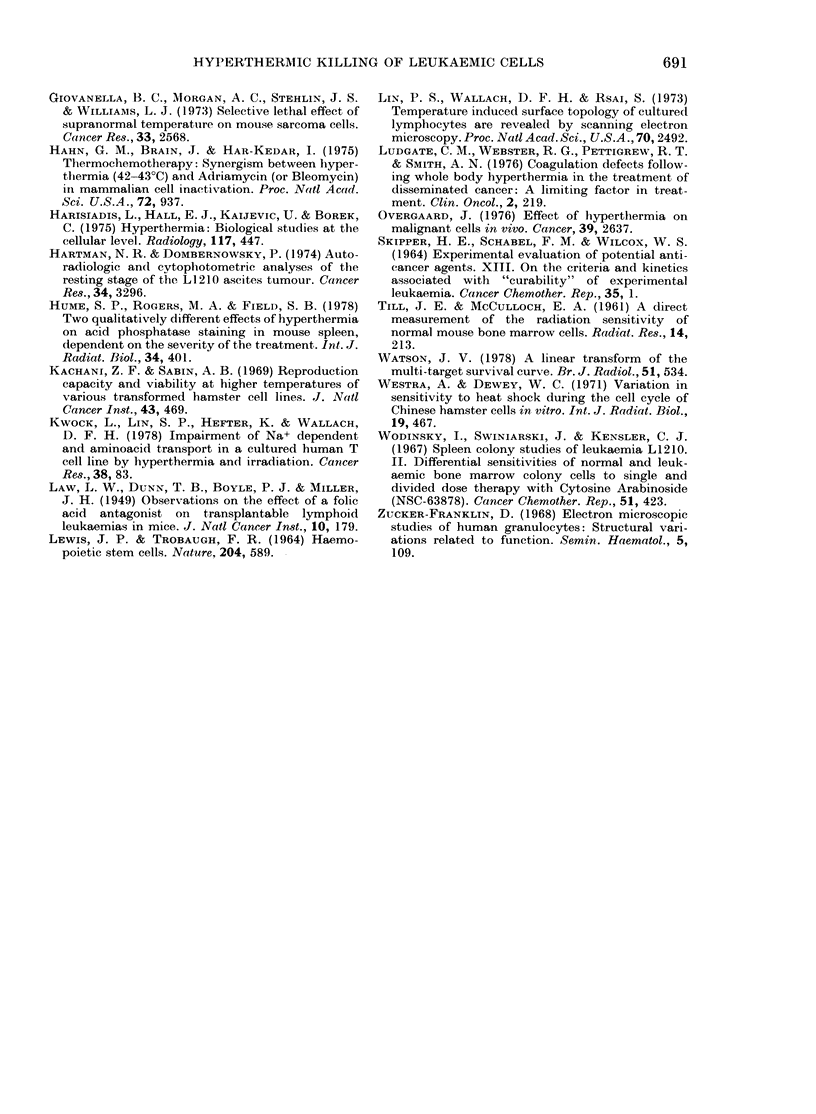# A comparison of the response to hyperthermia of murine haemopoietic stem cells (CFU-S) and L1210 leukaemia cells: enhanced killing of leukaemic cells in presence of normal marrow cells.

**DOI:** 10.1038/bjc.1981.254

**Published:** 1981-11

**Authors:** R. P. Symonds, T. E. Wheldon, B. Clarke, G. Bailey

## Abstract

**Images:**


*Br. J. Cancer* (1981) **44**, 682

### A COMPARISON OF THE RESPONSE TO HYPERTHERMIA OF MURINE HAEMOPOIETIC STEM CELLS (CFU-S) AND L1210 LEUKAEMIA CELLS: ENHANCED KILLING OF LEUKAEMIC CELLS IN PRESENCE OF NORMAL MARROW CELLS

R. P. SYMONDS, T. E. WHELDON*, B. CLARKE AND G. BAILEY†

*From the Radiobiology Research Group, Glasgow Institute of Radiotherapeutics and Oncology, Belvidere Hospital, Glasgow G32, the *MRC Cyclotron Unit, Hammersmith Hospital, London W12 0HS, and the ‡Dept. of Bio-Engineering, University of Strathclyde, Glasgow*

Received 9 February 1981 Accepted 23 July 1981

**Summary.—When the clonogenic survival of mouse haemopoietic stem cells (CFU-S) and leukaemia L1210 cells grown as ascites tumours are compared after being heated *in vitro* and assayed *in vivo* by spleen-colony assay, there is no significant difference in the terminal slopes of the survival curves. The shoulders of the survival curves differ, but this may be explained by differences in cell kinetics.**

**By contrast, L1210 leukaemic marrow cells are considerably more susceptible to the lethal effects of hyperthermia (43°C) than either normal marrow stem cells or L1210 leukaemic cells grown as ascites tumours. Moreover, the killing of L1210 ascites cells by hyperthermia can be enhanced by heating L1210 ascites cells with an equal number of normal marrow cells, or as upernatant removed from heated marrow cells.**

**Most cells in leukaemic marrow are normal, and it is postulated that the increased thermal sensitivity of L1210 cells in leukaemic marrow is caused by diffusible factors (*e.g.* lysosomal enzymes) released by heating normal marrow cells.**

THE POSSIBILITY that tumours may be destroyed by hyperthermia without unacceptable damage to surrounding tissues is the principal reason for the upsurge of interest in this treatment modality.

Both environmental and intrinsic differences between normal and tumour cells have been invoked as explanations for the favourable therapeutic differential seen when some solid tumours are treated by hyperthermia (Field & Bleehen, 1979).

Some workers (*e.g.* Giovanella *et al.*, 1973) have reported that malignant cells are inherently more sensitive to heat than their normal-tissue counterparts, whilst others have failed to find any difference in thermal sensitivity (*e.g.* Harisiadis *et al.*, 1975) or found normal cells more sensitive (*e.g.* Kachani, 1969). Interpretation of many such reports is, however, complicated by the variety of end points or assay systems used, and the attempted comparisons of normal and malignant cells of different tissue origin or even of different host species.

In this report we have attempted to avoid some of the problems associated with earlier studies by comparing the clonogenic survival of mouse haemopoietic stem cells (CFU-S) and L1210 leukaemia cells, growing in marrow or as an ascites tumour, heated *in vitro* and assayed *in vivo* by spleen-colony assay.

### MATERIALS AND METHODS

*Mice.*—DBA2 mice aged 10–16 weeks were used for all L1210 and most CFU-S hyperthermia survival experiments. C3H/He/Mg mice were used for a few CFU-S survival experiments to check whether results were strain-dependent.

*Medium.*—Cells were heated in Fischer's medium (Flow Labs) plus 10% (v/v) foetal calf serum and 2 mM L-glutamine added immediately before use. The Fischer's medium was stored at 4°C and the serum and L-glutamine were stored frozen. Medium age was found to be a significant factor, especially in the response of CFU-S (old medium giving lower survival) and in the experiments reported the medium used was not stored for more than 3 months.

*Tumour.*—L1210 leukaemia is a methylcholanthracene-induced lymphoblastic leukaemia (Law, 1949). Our strain was obtained from the Imperial Cancer Research Fund Laboratories in 1973 and had been stored in liquid $N_2$. After thawing, the cell line was maintained as an ascitic tumour by weekly passage in DBA2 mice.

*Normal tissue.*—When suitable numbers of marrow cells are injected into a supralethally irradiated mouse, discrete nodules are found in the spleen. Over 80% of the cells in each nodule are of one haemopoietic cell type (erythroid, granulocytic or megakaryocytic) (Lewis & Trobaugh, 1964). Each nodule has been shown to develop from one parent cell (Becker *et al.*, 1963), the colony-forming cell in spleen (CFU-S), believed to be the mouse haemopoietic pluripotential stem cell.

Up to 6 femurs were used to prepare a single-cell marrow suspension. If more than 6 femurs were used, the delay and increased manipulation decreased the viability of unheated control cells. On average $10^4$ unheated marrow cells had to be injected to produce one spleen colony.

*Preparation of leukaemic cell suspensions.*—L1210 cells were aspirated from the peritoneal cavities of frankly ascitic mice which had received an i.p. injection of $10^5$ cells 7 days earlier. Cells could easily be removed from non-ascitic mice 4 days after tumour inoculation by injecting 1 ml of warmed complete medium into the peritoneal cavity immediately before aspiration. Leukaemic marrow was prepared from the femurs of 3 mice killed by cervical dislocation 96 h after an i.v. injection of $10^6$ L1210 ascites cells. A single-cell suspension was produced by repeatedly sucking marrow gently through a 23-swg needle into a syringe. Cells were counted in a haemacytometer and diluted to experimental concentrations with complete medium.

*Hyperthermia technique.*—Sealed 10ml thin-wall glass tubes coated with silicone (Repelcote) containing 2 ml of cell suspension were heated in a Grant SX-35 thermostatically controlled waterbath (temperature variation ±0·01°C). Cell suspensions were kept at ice-water temperature before and after hyperthermia. When placed in the waterbath the temperature of the cell suspension was within 1°C of bath temperature within 90 sec and fully equilibrated to bath temperature in 195 sec. The time for the temperature within the tubes and the waterbath to equilibrate has not been subtracted from the experimental heating times, as the value is small and is partly balanced by the time taken for the tubes to cool.

In each experiment 4 tubes were heated at the same temperature, but each tube was removed from the bath at a different time. A control tube was left unheated at ice-water temperature. With minimal delay, aliquots from each tube were injected i.v. into groups of 4 mice. Twenty mice were used in each experiment.

*Spleen-colony assay of CFU-S.*—The method is essentially that of Till & McCulloch (1961). Recipient mice received a whole-body X-ray dose of 8·5 Gy (250 kVp—2·5 mmCu HVL) at a dose rate of 0·99 Gy/min. Nucleated marrow cells were injected i.v. within 3 h of irradiation. After 8 days the animals were killed by cervical dislocation and the spleens were removed and fixed in Bouin's solution. The macroscopic colonies on the surface of each spleen were counted.

*Spleen-colony assay of leukaemic clonogenic cells.*—The method is that of Wodinsky *et al.* (1967). Nucleated leukaemic marrow cells suspended in 0·2 ml of complete medium were injected i.v. into unirradiated DBA2 mice. After 6 days the animals were killed by cervical dislocation, the spleens removed and fixed in Bouin's solution. The number of macroscopic colonies on the surface of each spleen was counted. On average the injection of $10^2$ L1210 ascites cells or $10^3$ nucleated leukaemic marrow cells gave rise to 1 spleen colony.

*Preparation of scanning electron-microscope photographs.*—Aliquots of a suspension of L1210 ascites cells each containing $10^6$ cells suspended in 1 ml of complete medium were heated at 43°C for 5, 10, 15 or 30 min. Control aliquots of the same suspension were kept at 37°C, 4°C or room temperature (20–22°C) for 1 h. Cells were fixed by adding glutaraldehyde to 2·5% and dehydrated in graded concentrations of ethanol. Control and heated specimens were prepared identically. Gold-sputtered specimens were examined under a Philips 301 scanning electron microscope.

### RESULTS

### *Estimation of surviving fraction*

The cell-survival data were fitted using the multi-target expression:

$$S = 1 - (1 - \exp(-D/D_0))^n$$

where D is heating time at each fixed temperature and $D_0$ and n are parameters to be estimated for each data set. Estimation used was the method of Watson (1978) which linearizes the multi-target expression by a transformation, and fits the resultant straight line by the method of least squares. This method has the advantage that the fitting procedure makes use of all data points, including those on the shoulder.

A useful estimate of the width of the shoulder is provided by the $D_q$ value, given by $D_q = D_0 \ln(n)$.

### *Survival of L1210 (ascites) cells and CFU-S after hyperthermia*

Survival curves of L1210 cells heated over the temperature range 41–44°C suspended in Fischer's medium plus serum are shown in Fig. 1. Fig. 2 shows similar survival curves for DBA2 marrow CFU-S heated suspended in Fischer's medium supplemented with serum. Survival parameters for these experiments are listed in Table I.

Although the survival of L1210 ascites cells and CFU-S are very similar in the straight-line portion of the survival curves, the initial shoulders differ, the shoulder being much broader on the CFU-S curve.

### *Comparison of survival after hyperthermia of CFU-S from 2 mouse strains*

CFU-S from marrow removed from DBA2 or C3H/He/Mg mice were heated at 43°C in Fischer's medium plus serum. The mean $D_0$ of DBA2 CFU-S is 3·44 min (range 5·24–2·40 min) and for C3H CFU-S is 3·07 min (range 3·7–2·45.) The difference is clearly not significant.

FIG. 1.—Survival after hyperthermia of L1210 ascites cells suspended in Fischer's medium + serum.

### *Comparison of survival after hyperthermia (43°C) of L1210 cells from 7 and 4 day ascites*

Skipper *et al.* (1964) have demonstrated that leukaemic cells in the peritoneal cavity of a mouse are in the exponential phase of growth 4 days after an i.p. injection of $10^5$ L1210 cells. After 7 days, the

TABLE I.—*Survival parameters (min) after hyperthermia (41–44°C) for CFU-S and L1210 ascites cells*

| Temperature (°C) | L1210 ascites cells heated in Fischer's medium plus serum | | DBA2 marrow CFU-S heated in Fischer's medium plus serum | |
|---|---|---|---|---|
| | $D_0$ | $D_q$ | $D_0$ | $D_q$ |
| 41* | 30 | 10·1 | — | — |
| 42 | 16·08 | 1·76 | 16·15 | 17·34 |
| 43 | 4·98 | 1·47 | 3·45 | 7·8 |
| 44 | 2·05 | 0·15 | 1·44 | 4·00 |

* Since most of the data points after 41°C are in the shoulder region the numerical value may be imprecise.

FIG. 2.—Survival after hyperthermia of DBA2 marrow CFU-S suspended in Fischer's medium + serum.

FIG. 3.—Survival curves of L1210 cells from 4-day (open symbols broken line) and 7-day ascites (solid symbols entire line) after a hyperthermia treatment at 43°C.

rate of cell division slows and cells enter a decelerating phase of growth. The hyperthermic survival of L1210 cells in exponential and decelerating phase was studied by comparing the survival of 4 and 7 day ascites (Fig. 3). Survival parameters of these curves are listed in Table II.

*Survival after hyperthermia (43°C) of L1210 leukaemic cells of ascites and marrow origin*

The survival curves of L1210 cells of ascites (7-day) and marrow origin heated at 43°C are shown in Fig. 4. The survival of CFU-S is compared to that of L1210 cells from ascites and marrow in Fig. 5. Survival parameters derived from the curves are listed in Table II.

The very large difference between the heat sensitivity of L1210 cells of ascites or marrow origin is immediately apparent.

TABLE II.—*Survival parameters of CFU-S and L1210 cells from marrow and ascites following hyperthermia (43°C)*

| Cell type | $D_0$ (min) | $D_q$ (min) |
|---|---|---|
| L1210 cells from 7-day ascites | 4·98 | 1·57 |
| L1210 cells from 4-day ascites | 5·10 | 5·56 |
| L1210 cells from marrow | 1·1 | 5·67 |
| CFU-S | 3·45 | 7·8 |

*The survival after hyperthermia of a mixture of L1210 ascites cells and healthy marrow cells*

When leukaemic marrow receives hyperthermia, most of the heated cells are normal healthy marrow cells. In view of the different $D_0$ values of L1210 cells of marrow and ascitic origin (Table II) the contribution of normal marrow cells was studied by adding $10^7$ healthy nucleated marrow cells suspended in 1 ml of complete medium to the same number of L1210 ascites cells in the same volume of medium,

FIG. 4.—Comparison of survival after hyperthermia (43°C) of L1210 cells from ascites (solid symbols, entire line) and from leukaemic marrow (open symbols, broken line).

FIG. 5.—Comparison of survival after hyperthermia (43°C) of haemopoietic stem cells (CFU-S, ●) and L1210 cells from ascites (▲) or leukaemic marrow (×).

and immediately heating the mixture at 43°C for 15 min.

As shown in Fig. 6 the addition of nucleated marrow cells reduced the surviving fraction of L1210 cells by a factor of almost $10^{-2}$.

Control mice received $2 \times 10^3$ L1210 ascites cells with or without $10^6$ nucleated marrow cells. The addition of marrow cells made no difference to the number of spleen colonies produced.

*The survival of L1210 ascites cells heated with or without a cell-free supernatant from heated marrow*

A single-cell suspension was prepared from the marrow in 4 femurs removed from healthy DBA2 mice. After counting, the suspension was diluted to $10^7$ cells/ml with complete Fischer's medium and heated at 43°C for 15 min. Following centrifugation at 1000 rev/min for 10 min, the supernatant was removed and the cells discarded. Portions of the supernatant (each 1 ml) were added to tubes containing $10^6$ or $10^7$ L1210 ascites cells which were heated at 43°C for 7 and 20 min respectively. L1210 ascites cells from the same stock suspension were heated at the same time.

Control suspensions of L1210 ascites cells were stored at ice-water temperature with or without the cell-free supernatant. Similar numbers of spleen colonies were produced by both control specimens.

As shown in Fig. 7, the addition of heated marrow suspension decreased

FIG. 6.—Survival after hyperthermia (43°C) of L1210 ascites cells alone (solid symbols) and L1210 ascites cells mixed with an equal number of nucleated marrow cells (open symbols).

FIG. 7.—Survival after hyperthermia (43°C) of L1210 ascites cells alone (—) and with the supernatant from heated healthy marrow cells (---).

L1210 cell survival to the same extent as the addition of nucleated marrow cells. The effect is more pronounced when $10^6$ rather than $10^7$ L1210 cells are heated with 1 ml of the cell-free supernatant.

*The appearance of L1210 ascites cells examined by scanning electron microscopy after hyperthermia*

SEM failed to reveal any differences in the surface morphology of unheated L1210 ascites cells stored at 37°C or room temperature. Cells were seen as smooth spheres covered in microvilli (Fig. 8). Storage at 4°C induced minor changes in the microvilli which shortened and thickened.

No obvious changes were seen in cells heated for 5 and 10 min at 43°C. Although most cells heated for 15 min appeared normal, some had lost microvilli and appeared to have "bald areas". The surface morphology of all cells heated for 30 min was abnormal. Virtually all microvilli had disappeared and the normally smooth membrane was corrugated. Blebs or blisters appeared to have developed upon the surface of some cells (Fig. 9).

### DISCUSSION

These data provide information on the heat sensitivity of a normal cell type

FIG. 8.—Scanning electron micrograph showing an L1210 ascites cell and doughnut-shaped red cells kept at 37°C for 1 h before fixation. The L1210 cells membrane is smooth and covered in microvilli (line marker = 1 $\mu$m).

FIG. 9.—Scanning electron micrograph of an L1210 ascites cell heated at 43°C for 30 min. Microvilli have been lost from the L1210 cell surface and the cell membrane is blistered. A polymorph leucocyte and red cells can be seen in the foreground (line marker = 1 $\mu$m).

(CFU-S) with clonogenic capability, and of the nearest-equivalent neoplastic cell type, a leukaemic clonogenic cell. In view of the clinical potential of hyperthermia, and of the conflicting evidence on the "intrinsic" heat sensitivity of normal and malignant cells (see Field & Bleehen, 1979), it is of interest to compare the heat sensitivities of these cell types. However, comparison of the heat sensitivity of CFU-S with that of L1210 ascites cells, or of CFU-S with L1210 marrow cells lead to different conclusions.

These results provide no indication of significant differences in the thermal sensitivity of normal marrow stem cells and of leukaemic clonogenic cells grown in an ascites environment. No significant differences between the terminal slopes of the survival curves of the normal and leukaemic cells were found.

Differences do exist in the shoulder region, however, normal cells displaying consistently higher $D_q$ than leukaemic cells. This result may possibly be explicable in terms of cell kinetics.

Normally, most CFU-S are in the resting ($G_0$) phase of the cell cycle (Becker *et al.*, 1965) whilst, 7 days after an i.p. inoculum of $10^5$ cells, most L1210 cells, though in the decelerating growth phase, are still actively cycling (Hartman *et al.*, 1974). For CHO cells, Westra & Dewey (1971) found the heat-survival curve slope to be similar for cells in all phases of the cell cycle, but cells heated in M or S had a reduced shoulder width.

The $D_0$ values of CFU-S and L1210 in this study are the lowest yet recorded for any cell line subjected to hyperthermia. Bhuyan (1979) reported a $D_0$ of 10 min for L1210 cells heated at 43°C and assayed *in vitro* by cloning in soft agar. The different assay technique may account for our different results.

By contrast, L1210 cells from marrow are 4 times as sensitive to heat as L1210 ascites cells or CFU-S. This difference does not appear to be caused by cell-kinetic dissimilarities. Although exponential phase (4-day) ascites and decelerating phase (7-day) ascites have markedly different cell and population kinetics (Dombernowsky & Hartman, 1972) the $D_0$ values of the 2 survival curves (Table II) are not significantly different. The broader shoulder of the 4-day ascites curve suggests that 4-day ascites cells have a greater capacity to accumulate or repair sublethal damage than 7-day cells, possibly because of the better nutritional environment of cells during exponential growth.

Only a small percentage of nucleated cells within leukaemic marrow are L1210 cells. As the addition of an equal number of healthy marrow cells markedly enhances the lethal effect of hyperthermia on L1210 ascites cells (Fig. 6) the increased thermal sensitivity of L1210 leukaemic marrow cells is probably the result of the many normal marrow cells in the heated suspension. Sensitization of L1210 leukaemic cells by normal marrow does not appear to be a direct interaction between intact cells, as increased hyperthermic killing is proportional to the amount of cell-free supernatant added to the L1210 cell suspension.

The broad shoulder of the L1210 leukaemic curve (Fig. 3) may correspond to the time taken for a sensitizing process to be initiated. On this interpretation the survival curves of L1210 ascites and leukaemic marrow would be identical for the first 10 min of heating, but the curves would then diverge. This interpretation seems consistent with the observations. It is possible that enhanced killing of L1210 cells in leukaemic marrow only occurs as normal marrow cells are damaged and diffusible substances are released. Such a mechanism would lead to the rather large $D_q$ but small $D_0$ observed for L1210 cells in a marrow environment.

One possibility is that the substances released from heated marrow cells are lysosomal enzymes. Several authors (*e.g.* Overgaard, 1976) have suggested that lysosomes may be important targets for thermal damage *in vivo*. It has been shown (Hume *et al.*, 1978) that hyperthermia increases splenic lysosomal acid phosphatase activity and the permeability of lysosomal membranes.

Of course, lysosomal enzymes would not normally penetrate the intact membrane of an adjacent cell, but the membranes of heat-damaged cells may not be intact. The surface morphology of L1210 cells is grossly altered by heat, with loss of microvilli, and corrugation and blistering of the cell surface (see Fig. 8). Similar morphological changes have been described by Lin *et al.* (1973) for NBC-6 lymphocytes exposed to 45°C, whilst Kwock *et al.* (1978) have correlated the surface changes of heated lymphoid cells with membrane permeability changes and inhibition of the sodium pump. In addition, Hahn *et al.* (1975) have reported heat-induced permeability changes which allow increased quantities of cytotoxic drugs to enter the cell.

We postulate that the increased killing of L1210 cells which occurs when the cells are heated in presence of marrow is caused by a release from heat-damaged cells of lysosomal enzymes capable of penetrating the membranes of adjacent cells rendered unusually permeable by heat. Apparently, the leukaemic cells are more susceptible to this secondary damage than normal marrow stem cells, resulting in the observed differential response to heat of normal and leukaemic cells (see Fig. 3).

Though similar phenomena might occur with other cell types, the release of lysosomal enzymes from heat-damaged cells may be especially important for marrow. About half the volume of a mature neutrophil is occupied by up to 600 lysosomal granules, each 0·5 μm in diameter (Zucker-Franklin, 1968). Enzymes within the granules include acid and alkaline phosphatases, nucleotidase, deoxyribonuclease and β-glucuronidase (Ackerman, 1964).

The release of neutrophil lysosomal enzymes into tissues enhances or perpetuates any existing inflammatory response whatever the cause. Moreover, during the remission induction of acute promyelocytic leukaemia, neoplastic cells killed by cytotoxic drugs release lysosomal enzymes which may precipitate severe disseminated intravascular coagulation (Bernard *et al.*, 1973). A similar mechanism could underlie the occurrence of disseminated intravascular coagulation following whole-body hyperthermia in human patients (Ludgate *et al.*, 1976) and could be an important form of toxicity associated with this treatment.

Whatever the mechanisms responsible for the sensitizing effect of marrow on L1210 leukaemia cells, the possibility that cell–cell interactions may be implicated in hyperthermia responses deserves further consideration.

If the differential heat sensitivity of normal and leukaemic cells in marrow proves to be a general phenomenon, it may be clinically exploitable. One avenue might be the use of hyperthermia to eliminate small numbers of malignant cells from stored autologous marrow removed from patients with leukaemia or (possibly) disseminated solid tumour.

However, it will be necessary to check the generality of the favourable differential sensitivity, using different leukaemias, and to confirm that observed differences in apparent cell survival do not result from reduced ability of leukaemic cells to lodge in the spleens of recipient mice, whilst lodging normally, and retaining clonogenicity, in other sites.

This latter possibility could be tested experimentally by a "lifespan assay", instead of spleen-colony assay, to confirm that the sensitizing effect on leukaemic cells of normal marrow alters the total number of cells retaining clonogenic capacity, in any anatomical site.

### CONCLUSIONS

(1) There exists no significant difference between the $D_0$ times for survival of normal marrow cells (CFU-S) and leukaemic (L1210) clonogenic ascites cells, within the temperature range 42–44°C.

(2) Differences in shoulder width do exist, the normal cells having the wider shoulder, but this may be explicable in terms of cell kinetics.

(3) L1210 leukaemic cells of marrow origin are much more susceptible to the lethal effects of hyperthermia at 43°C than L1210 ascites cells or haemopoietic stem cells.

(4) The killing of L1210 ascites cells by hyperthermia can be enhanced by heating L1210 ascites cells with an equal number of healthy marrow cells or a supernatant removed from heated normal marrow cells.

(5) When examined under the scanning electron microscope L1210 ascites cells subjected to hyperthermia show gross morphological changes such as corrugation and blistering of the cell-membrane and loss of microvilli. These changes in the appearance of the cell are similar to those associated with changes in cell-membrane permeability.

(6) It is possible that the enhanced killing of L1210 leukaemic cells by hyperthermia in the presence of normal marrow cells is caused by lysosomal enzymes released from healthy marrow cells.

(7) Cell–cell interactions involving the release of injurious substances from heat-damaged cells may constitute another type of environmental modulation of cellular response to hyperthermia which is additional to, and perhaps independent of, other environmental influences (*e.g.* nutrition, pH) already recognized to be of importance.

We are grateful for the help and advice of Dr N. G. L. Harding, Professor A. H. W. Nias and Dr E. H. Porter.

During this study R. P. Symonds was in receipt of an MRC Research Training Fellowship.

### REFERENCES

Ackerman, G. A. (1964) Histochemical differentiation during neutrophil development and maturation. *Ann. N.Y. Acad. Sci.*, **113**, 537.

Becker, A. J., McCulloch, E. A. & Till, J. E. (1963) Cytological demonstration of the clonal nature of spleen colonies derived from transplanted mouse marrow cells. *Nature*, **197**, 452.

Becker, A. J., McCulloch, E. A., Siminovith, L. & Till, J. E. (1965) The effect of differing demands for blood cell production on DNA synthesis by haemopoietic colony forming cells in mice. *Blood*, **26**, 296.

Bhuyan, B. K. (1979) Kinetics of cell kill by hyperthermia. *Cancer Res.*, **39**, 2277.

Bernard, J., Wei, I. M., Boiron, M., Jacquillat, C., Flandrun, G. P Gemon, M. F. (1973) Acute promyelocytic leukaemia: Results of treatment by Daunorubicin. *Blood*, **41**, 489.

Dombernowsky, P. & Hartman, N. R. (1972) Analysis of variation in the cell population kinetics with tumour age in the L1210 ascites tumour. *Cancer Res.*, **32**, 2452.

Field, S. B. & Bleehen, N. M. (1979) Hyperthermia in the treatment of cancer. *Cancer Treat. Rev.*, **6**, 63.

GIOVANELLA, B. C., MORGAN, A. C., STEHLIN, J. S. & WILLIAMS, L. J. (1973) Selective lethal effect of supranormal temperature on mouse sarcoma cells. *Cancer Res.*, **33**, 2568.

HAHN, G. M., BRAIN, J. & HAR-KEDAR, I. (1975) Thermochemotherapy: Synergism between hyperthermia (42–43°C) and Adriamycin (or Bleomycin) in mammalian cell inactivation. *Proc. Natl Acad. Sci. U.S.A.*, **72**, 937.

HARISIADIS, L., HALL, E. J., KAIJEVIC, U. & BOREK, C. (1975) Hyperthermia: Biological studies at the cellular level. *Radiology*, **117**, 447.

HARTMAN, N. R. & DOMBERNOWSKY, P. (1974) Autoradiologic and cytophotometric analyses of the resting stage of the L1210 ascites tumour. *Cancer Res.*, **34**, 3296.

HUME, S. P., ROGERS, M. A. & FIELD, S. B. (1978) Two qualitatively different effects of hyperthermia on acid phosphatase staining in mouse spleen, dependent on the severity of the treatment. *Int. J. Radiat. Biol.*, **34**, 401.

KACHANI, Z. F. & SABIN, A. B. (1969) Reproduction capacity and viability at higher temperatures of various transformed hamster cell lines. *J. Natl Cancer Inst.*, **43**, 469.

KWOCK, L., LIN, S. P., HEFTER, K. & WALLACH, D. F. H. (1978) Impairment of $Na^+$ dependent and aminoacid transport in a cultured human T cell line by hyperthermia and irradiation. *Cancer Res.*, **38**, 83.

LAW, L. W., DUNN, T. B., BOYLE, P. J. & MILLER, J. H. (1949) Observations on the effect of a folic acid antagonist on transplantable lymphoid leukaemias in mice. *J. Natl Cancer Inst.*, **10**, 179.

LEWIS, J. P. & TROBAUGH, F. R. (1964) Haemopoietic stem cells. *Nature*, **204**, 589.

LIN, P. S., WALLACH, D. F. H. & RSAI, S. (1973) Temperature induced surface topology of cultured lymphocytes are revealed by scanning electron microscopy. *Proc. Natl Acad. Sci., U.S.A.*, **70**, 2492.

LUDGATE, C. M., WEBSTER, R. G., PETTIGREW, R. T. & SMITH, A. N. (1976) Coagulation defects following whole body hyperthermia in the treatment of disseminated cancer: A limiting factor in treatment. *Clin. Oncol.*, **2**, 219.

OVERGAARD, J. (1976) Effect of hyperthermia on malignant cells *in vivo*. *Cancer*, **39**, 2637.

SKIPPER, H. E., SCHABEL, F. M. & WILCOX, W. S. (1964) Experimental evaluation of potential anticancer agents. XIII. On the criteria and kinetics associated with "curability" of experimental leukaemia. *Cancer Chemother. Rep.*, **35**, 1.

TILL, J. E. & MCCULLOCH, E. A. (1961) A direct measurement of the radiation sensitivity of normal mouse bone marrow cells. *Radiat. Res.*, **14**, 213.

WATSON, J. V. (1978) A linear transform of the multi-target survival curve. *Br. J. Radiol.*, **51**, 534.

WESTRA, A. & DEWEY, W. C. (1971) Variation in sensitivity to heat shock during the cell cycle of Chinese hamster cells *in vitro*. *Int. J. Radiat. Biol.*, **19**, 467.

WODINSKY, I., SWINIARSKI, J. & KENSLER, C. J. (1967) Spleen colony studies of leukaemia L1210. II. Differential sensitivities of normal and leukaemic bone marrow colony cells to single and divided dose therapy with Cytosine Arabinoside (NSC-63878). *Cancer Chemother. Rep.*, **51**, 423.

ZUCKER-FRANKLIN, D. (1968) Electron microscopic studies of human granulocytes: Structural variations related to function. *Semin. Haematol.*, **5**, 109.